# The value of intravoxel incoherent motion model-based diffusion-weighted imaging for predicting long-term outcomes in nasopharyngeal carcinoma

**DOI:** 10.3389/fonc.2022.902819

**Published:** 2022-12-01

**Authors:** Yuhui Qin, Chen Chen, Haotian Chen, Fabao Gao

**Affiliations:** Department of Radiology, West China Hospital, Sichuan University, Chengdu, China

**Keywords:** nasopharyngeal carcinoma, chemoradiotherapy, prognosis, intravoxel incoherent motion, diffusion-weighted imaging, long-term outcome

## Abstract

**Objective:**

The aim of this study was to evaluate the prognostic value for survival of parameters derived from intravoxel incoherent motion diffusion-weighted imaging (IVIM-DWI) in patients with nasopharyngeal carcinoma (NPC).

**Materials:**

Baseline IVIM-DWI was performed on 97 newly diagnosed NPC patients in this prospective study. The relationships between the pretreatment IVIM-DWI parametric values (apparent diffusion coefficient (ADC), D, D*, and f) of the primary tumors and the patients’ 3-year survival were analyzed in 97 NPC patients who received chemoradiotherapy. The cutoff values of IVIM parameters for local relapse-free survival (LRFS) were identified by a non-parametric log-rank test. The local-regional relapse-free survival (LRRFS), LRFS, regional relapse-free survival (RRFS), distant metastasis-free survival (DMFS), progression-free survival (PFS), and overall survival (OS) rates were calculated by using the Kaplan–Meier method. A Cox proportional hazards model was used to explore the independent predictors for prognosis.

**Results:**

There were 97 participants (mean age, 48.4 ± 10.5 years; 65 men) analyzed. Non-parametric log-rank test results showed that the optimal cutoff values of ADC, D, D*, and f were 0.897 × 10^−3^ mm^2^/s, 0.699 × 10^−3^ mm^2^/s, 8.71 × 10^−3^ mm^2^/s, and 0.198%, respectively. According to the univariable analysis, the higher ADC group demonstrated significantly higher OS rates than the low ADC group (*p* = 0.036), the higher D group showed significantly higher LRFS and OS rates than the low D group (*p* = 0.028 and *p* = 0.017, respectively), and the higher D* group exhibited significantly higher LRFS and OS rates than the lower D* group (*p* = 0.001 and *p* = 0.002, respectively). Multivariable analyses indicated that ADC and D were the independent prognostic factors for LRFS (*p* = 0.041 and *p* = 0.037, respectively), D was an independent prognostic factor for LRRFS (*p* = 0.045), D* and f were the independent prognostic factors for OS (*p* = 0.019 and 0.029, respectively), and f acted was an independent prognostic factor for DMFS (*p* = 0.020).

**Conclusions:**

Baseline IVIM-DWI perfusion parameters ADC and D, together with diffusion parameter D*, could act as useful factors for predicting long-term outcomes and selecting high-risk patients with NPC.

## Introduction

Nasopharyngeal carcinoma (NPC) is an aggressive tumor that is common in Southern China and Southeast Asia (1). Unlike most other head and neck cancer, which are mainly treated by surgery, definitive radiotherapy and concurrent chemoradiotherapy are the primary treatment for NPC (2–4). With the application of advanced radiation therapy equipment and multimodal therapy, the 5-year overall survival rate of NPC has improved obviously with a range from 75% to 83.5% according to the latest studies from Southern China (5, 6). However, local residue and recurrence remain the main reasons for treatment failure for NPC (7, 8). Therefore, the management of recurrent NPC remains a challenging clinical problem. Predicting the prognosis for chemoradiotherapy may provide helpful information to improve the therapeutic regimen, so it is important to identify biomarkers that are valuable to predict outcomes and provide more accurate information on the selection of the most effective treatment strategy for NPC.

Nowadays, magnetic resonance imaging (MRI) plays an important role in managing NPC patients such as clinical staging, detecting lesions, and evaluating therapy effects (9–12). Convention morphology-based MRI provides little benefit in the prediction of treatment effects in NPC. Diffusion-weighted imaging (DWI) has the ability to measure the motion of water molecules by calculating the apparent diffusion coefficient (ADC) value; thus, it can quantitatively analyze the tissue microstructure (11). Intravoxel incoherent motion diffusion-weighted imaging (IVIM-DWI) is an advanced DWI technique based on the bi-exponential model (13), which can simultaneously obtain diffusion and perfusion information in tissues. Previous studies have shown the potential of IVIM-DWI in the evaluation of head and neck cancer (11, 14–16). Most of these studies have focused on estimating therapeutic effects within 6 months (11, 14, 17), and few reports have assessed the usefulness of IVIM-DWI in predicting the long-term outcome in NPC. Thus, the present study was conducted to evaluate the utility of IVIM-DWI parameters derived from the primary lesion in predicting the long-term outcome for NPC.

## Methods and materials

### Patient selection and treatment

This prospective study was approved by the Medical Ethics Committee of our institution. Informed consent documents were signed by all participants. Patient inclusion criteria were as follows: a) was diagnosed with NPC and had pathological confirmation of NPC, b) age ≥ 18 years, and c) had plans for chemoradiotherapy. Patient exclusion criteria were as follows: a) had previously undergone anti-tumor therapy for NPC; b) did not sign the informed consent; c) had contraindications for MRI, chemotherapy, or radiotherapy; d) allergic to gadolinium contrast media. Patients were eliminated if a) their imaging quality was poor, b) they dropped out of treatment, c) intensity-modulated radiotherapy (IMRT) was discontinued for more than 1 week, or d) they received anti-tumor treatment other than chemoradiotherapy.

The primary tumor (T-staging) and nodal metastases (N-staging) were determined according to the 8th edition of the Union for International Cancer Control/American Joint Committee on Cancer (UICC/AJCC) staging system (18). All patients underwent contrast-enhanced MRI on the neck and nasopharynx, chest X-ray, chest CT, liver ultrasonography or CT scan, and a whole-body single-photon emission computed tomography (SPECT) bone imaging, together with a complete medical history, physical checkup, hematology, and serum biochemistry profiles for baseline evaluation. Those patients at risk of distant metastases received a whole-body ^18^F-fluorodeoxyglucose positron emission tomography (PET)/CT. A biopsy of the primary lesion before treatment was performed to determine pathological types for NPC. Patients received IMRT combined with induction, concurrent, or adjuvant chemotherapy according to their clinical stage.

All patients received IMRT for their primary and neck lesions. In total, 87 patients with stage II–IVa/b were treated with platinum-based induction chemotherapy + chemoradiotherapy, concurrent chemoradiotherapy, or concurrent chemoradiotherapy + adjuvant chemotherapy. Ten cases with stage IVc underwent platinum-based doublet chemotherapy for 3 to 6 cycles or until the presence of disease progression, death, or intolerable toxicities or at the patients’ request to cease.

### MRI scans

The MRI examinations were performed on a 1.5-Tesla MRI scanner (Optima MR360, GE Healthcare, Milwaukee, WI, USA) at baseline using a head and neck coil. The imaging protocols included axial T1-weighted spin-echo images (repetition time (TR) = 580 ms, echo time (TE) = 7.8 ms, field of view (FOV) = 38 cm, slice thickness = 5 mm, slice space = 1 mm, slice number = 36, number of excitations (NEX) = 2) and axial T2- weighted fast spin-echo images (TR = 6,289 ms, TE = 85 ms, FOV = 38 cm, slice thickness = 5 mm, slice space = 1 mm, slice number = 36, NEX = 2).

### Intravoxel incoherent motion diffusion-weighted imaging protocol

After conventional MRI scans, IVIM-DWI was performed using a single-shot diffusion-weighted spin-echo echo-planar (SS-SE-DW-EPI) sequence with 10 different b values (0, 50, 80, 100, 150, 200, 400, 600, 800, and 1,000 s/mm^2^). Multifarious b values were measured in one series by adjusted lookup table of gradient directions. Parallel imaging with an acceleration factor of 2 was applied. The susceptibility artifacts were minimized by a local shim box covering the nasopharynx. Twelve axial slices covering the nasopharynx were obtained using the following parameters: FOV = 22 cm, slice thickness = 5 mm, slice space = 1 mm, TR/TE = 4,225/106 ms, matrix = 128 × 130, NEX = 4, and scan time = 2 min 55 s.

### Data analysis

All the MRI data were transmitted to an Advantage Workstation (version AW 4.6; GE Medical Systems, Milwaukee, WI, USA) for postprocessing. IVIM analysis was performed using a MADC Kit, and four parameters derived from IVIM-DWI (ADC; pure diffusion coefficient, D; pseudo-diffusion coefficient, D*; perfusion fraction, f) of each primary NPC lesion were generated on the basis of a pixel-by-pixel fitting according to the Levenberg–Marquardt algorithm (19). The IVIM-DWI parametric values for each tumor were measured by two radiologists (A and B; both had more than 10 years of experience in head and neck radiology) independently and double-blindly. To measure the four IVIM-DWI parametric values, the axial image covering the widest cross-section of the primary tumor was determined using the T2-weighted and contrast-enhanced T1-weighted images as references. Then free-hand regions of interest (ROIs) were delineated on the axial T2-weighted images by each observer for each tumor at its widest section to cover as much of the nasopharyngeal tumor as possible but avoiding the areas of necrosis, air, large vessels, and adjacent anatomical structures (i.e., fat, muscle, and bone). To avoid volume averaging, the most superior and inferior slices for each tumor were excluded. According to all the ROIs of the tumor, a three-dimensional (3D) volume of interest (VOI) for this tumor was generated by the MADC kit automatically and then output the mean ADC value of this VOI. Other IVIM-DWI maps were evaluated as the same, and the corresponding parametric values were generated.

### Clinical endpoint

Patients received regular clinical and endoscopic examinations every 3 months in the first 2 years and every 6 months for the next 3 years or until death. General examination (clinical symptoms and physical examination) and imaging examination were performed for the follow-up of all patients. Local recurrence was determined based on pathological diagnosis (endoscopic biopsy) or MRI findings (detection of a new nasopharyngeal mass, continuous increase in tumor size, or progressive erosion of the skull base bone). Any cervical lymph node recurrence detected by physical examination, needle aspiration biopsy, and/or MRI exam on the neck was regarded as a regional relapse. Local-regional relapse was defined as a patient who had both local and regional recurrence. Distant failure was defined as the detection of metastases in any location (except for the cervical node) such as in the bone, liver, lung, or mediastinum. If necessary, PET/CT would be performed to determine relapse or metastases for doubtful cases.

### Statistical analysis

Local-regional relapse-free survival (LRRFS), local relapse-free survival (LRFS), regional relapse-free survival (RRFS), distant metastasis-free survival (DMFS), progression-free survival (PFS), and overall survival (OS) were the survival endpoints in the present study. LRRFS, LRFS, RRFS, DMFS, and OS were respectively defined as the period from the initial day of therapy to the first local-regional relapse, local recurrence, regional relapse, distant metastasis, and death. PFS was measured from the first day of treatment to disease progression or death from any cause (20, 21).

SPSS version 22.0 (SPSS Inc., Chicago, IL, USA) was used for statistical analyses. *p* < 0.05 was considered significant. The interclass correlation coefficient (ICC) was used to evaluate the interobserver agreement for IVIM parameters and was interpreted as follows: 0.21–0.40, fair correlation; 0.41–0.60, moderate correlation; 0.61–0.80, good correlation; 0.81–1.00, excellent correlation (22). The cutoff values of IVIM parameters for LRFS were identified by a non-parametric log-rank test. The Kaplan–Meier estimator with the log-rank test was utilized to calculate the survival rate in order to compare the differences between groups. To identify independent predictors for the outcome, significant IVIM-DWI- derived parameters, together with confounding factors (gender, age, clinical stage, treatment regimen, and pathological type), were selected as input variables in subsequent multivariate logistic regression analysis based on the Cox proportional hazards model to investigate their associations with survival.

## Results

### Study participants

**Figure 1:**
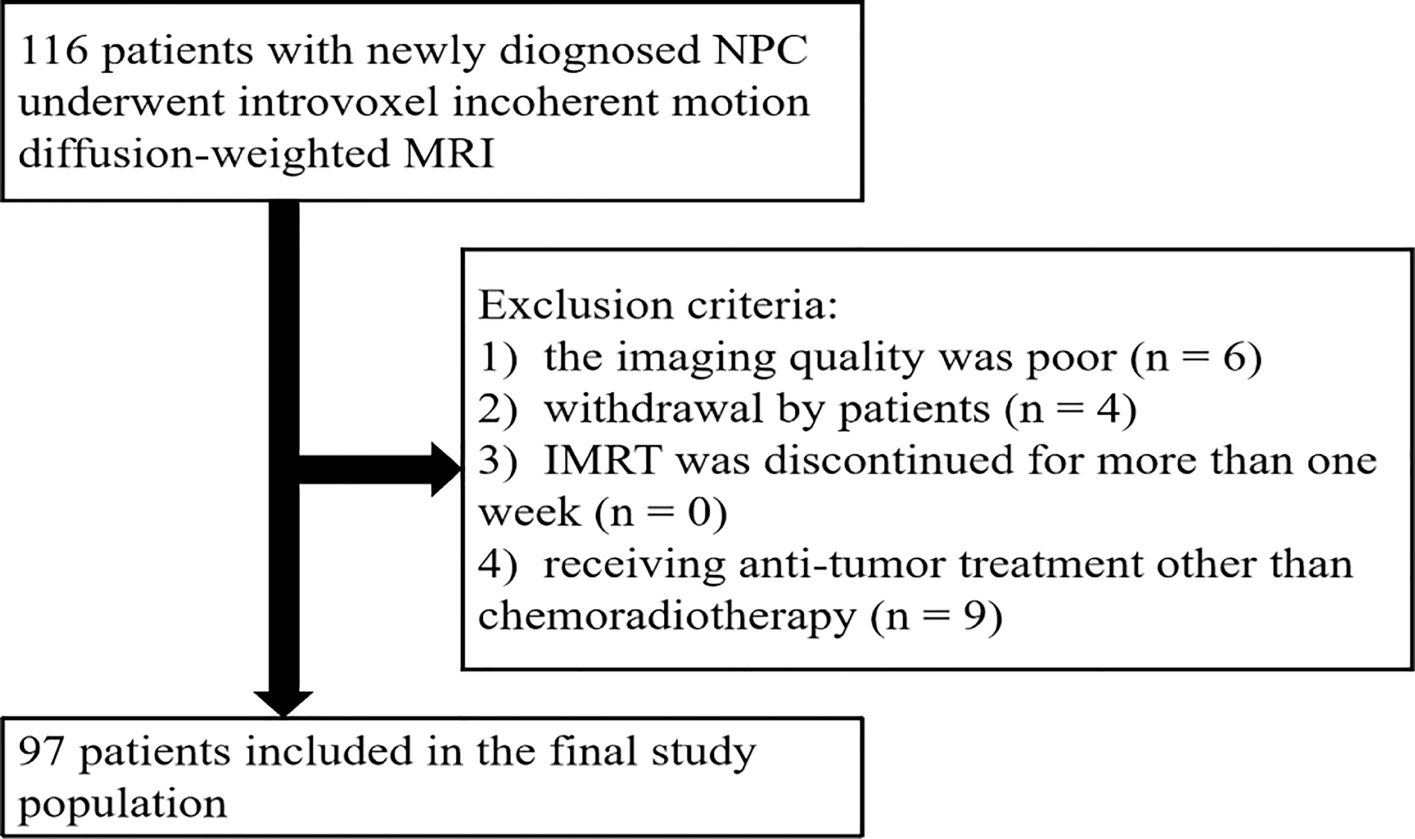
The flowchart of patient inclusion and exclusion.

From November 2016 to May 2018, a total of 116 patients were initially enrolled. The flowchart shows the initial number of patients, patients excluded, and the final study population in this study (**Figure 1**). Finally, 97 patients (48.4 ± 10.5 years [standard deviation]; age range, 26–67 years; 65 men and 32 women) were enrolled, with clinical stages of II–IV. The mean follow-up period for the 97 NPC patients was 38.7 months (range, 8–49 months). During the follow-up period, eight patients were lost to follow-up, and their survival time was 8–19 months. The clinical characteristics of the patient cohort in this study are shown in **Table 1**.

**Table 1:** Clinical and imaging features of the 97 patients with nasopharyngeal carcinoma.

Characteristic	Result	*p*
Sex		0.120
Men	65 (67.0%)	
Women	32 (33.0%)	
Mean age (years)		0.122
All patients	48.4 ± 10.5	
Men	49.0 ± 10.0	
Women	47.1 ± 11.5	
T stage		0.658
T1	12 (12.3%)	
T2	33 (33.9%)	
T3	26 (26.9%)	
T4	26 (26.9%)	
N stage		0.829
N0	4 (4.1%)	
N1	12 (12.3%)	
N2	57 (58.8%)	
N3	24 (24.8%)	
M stage		0.819
M0	93 (95.8%)	
M1	4 (4.2%)	
Overall stage		0.385
II	8 (8.2%)	
III	43 (44.3%)	
IV	46 (47.5%)	
Pathological type		0.695
Keratinizing	6 (6.2%)	
Differentiated non-keratinizing	72 (74.2%)	
Undifferentiated non-keratinizing	19 (19.6%)	
Treatment regimen		0.186
Chemoradiotherapy	87 (89.7%)	
Chemotherapy	4 (4.1%)	
Radiotherapy	6 (6.2%)	
Median follow-up (months)†	38 (8-49)	
Mean IVIM-DWI parameters		
ADC‡	0.960 ± 0.202	0.062
≤0.897 × 10^−3^ mm^2^/s	0.792 ± 0.068	
>0.897 × 10^−3^ mm^2^/s	1.077 ± 0.180	
D‡	0.720 ± 0.181	0.028
≤0.699 × 10^−3^ mm^2^/s	0.568 ± 0.089	
>0.699 × 10^−3^ mm^2^/s	0.827 ± 0.149	
D*‡	18.824 ± 17.379	0.001
≤8.71 × 10^−3^ mm^2^/s	7.044 ± 1.882	
>8.71 × 10^−3^ mm^2^/s	24.356 ± 18.633	
f (%)‡	0.278 ± 0.092	0.257
≤0.198	0.164 ± 0.025	
>0.198	0.315 ± 0.074	
Status		
Local relapse	7 (7.2%)	
Non-local relapse	90 (92.8%)	

Data are the number of participants, and data in parentheses are percentages. Mean data are ± standard deviation.

IVIM-DWI, intravoxel incoherent motion diffusion-weighted imaging; ADC, apparent diffusion coefficient.

^†^Data in parentheses are range.

^‡^Data are dichotomized by using a non-parametric log-rank test.

### Clinical and intravoxel incoherent motion diffusion-weighted imaging findings

**Table 1** shows the clinical features of the 97 patients and their demographics. All the clinical factors were not associated with LRFS in the study; their *p*-value, hazard ratio (HR), and 95% confidence interval (CI) were as follows: sex, HR, 3.271; 95% CI: 0.734–14.576, *p* = 0.120; age, HR, 1.057; 95% CI: 0.985–1.135, *p* = 0.122; T stage, HR, 1.711; 95% CI: 0.158–18.491, *p* = 0.658; N stage, HR, 0.770; 95% CI: 0.072–8.284, *p* = 0.829; M stage, HR, 0; 95% CI: 0–0, *p* = 0.819; overall stage, HR, 0.385; 95% CI: 0.020–7.445, *p* = 0.528; pathological type, HR, 0.694; 95% CI: 0.111– 4.317, *p* = 0.695; treatment regimen, HR, 0.109; 95% CI: 0.004–2.916, *p* = 0.186 (**Table 1**). The ICC values of ADC, D, D*, and f were 0.82 (95% CI: 0.79–0.96), 0.94 (95% CI: 0.73–0.98), 0.85 (95% CI: 0.41–0.94), and 0.71 (95% CI: 0.61–0.90), respectively, indicating that the consistency of the data from the two observers is good. Therefore, the IVIM-DWI data obtained by the first observer were calculated in this study. The average values of ADC, D, D*, and f in the selected ROI were 0.960 × 10^−3^ ± 0.202 mm^2^/s, 0.720 × 10^−3^ ± 0.180 mm^2^/s, 18.823 × 10^−3^ ± 17.379 mm^2^/s, and 0.278% ± 0.092%, respectively (**Table 1**). Spearman’s rank correlation coefficient showed that ADC was correlated with D (Spearman’s r = 0.631, *p* = 0.012), and there was no correlation between ADC and D* (Spearman’s r = 0.149, *p* = 0.144), ADC and f (Spearman’s r = 0.126, *p* = 0.222), D and D* (Spearman’s r = 0.008, *p* = 0.940), D and f (Spearman’s r = 0.097, *p* = 0.349), and D* and f (Spearman’s r = 0.107, *p* = 0.298), as shown in **Table 2**.

**Table 2:** Correlations between ADC, D, D*, and f of IVIM-DWI parameters.

Parameter	Spearman’s rank correlation coefficient (Spearman’s r)	*p*
**ADC and D**	0.631	0.012
ADC and D*	0.149	0.144
**ADC and f**	0.126	0.222
D and D*	0.008	0.940
**D and f**	0.097	0.349
D* and f	0.107	0.298

IVIM-DWI, intravoxel incoherent motion diffusion-weighted imaging; ADC, apparent diffusion coefficient. *p<0.05.

### Intravoxel incoherent motion diffusion-weighted imaging parameters for predicting survival outcomes

**Figure 2:**
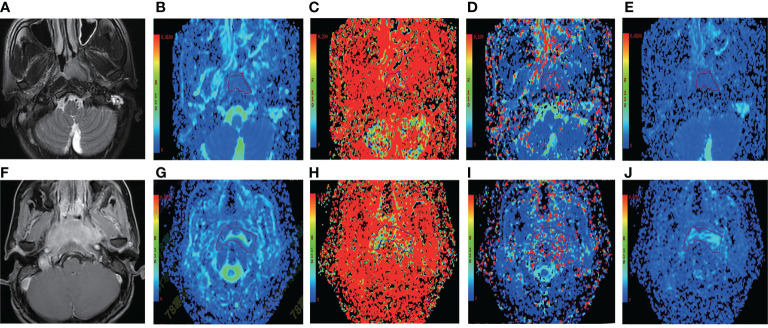
Representative T2WI and IVIM-DWI images from two NPC patients with different outcomes. **(A–E)** Images of a patient (case A) who suffered local failure. **(A)** Pretreatment T2WI. **(B–E)** Pretreatment ADC, f, D*, and D maps, respectively. The ADC, f, D*, and D values of the nasopharyngeal tumor in case A were 0.666 × 10^−3^ mm^2^/s, 0.354%, 3.78 × 10^−3^ mm^2^/s, and 0.581 × 10^−3^ mm^2^/s, respectively. The bottom row shows images from a patient (case B) who did not suffer local failure. **(F)** Pretreatment T2WI. **(G–J)** P retreatment ADC, f, D*, and D maps. The ADC, f, D*, and D values of the nasopharyngeal tumor in case B were 1.24 × 10^−3^ mm^2^/s, 0.192%, 17.9 × 10^−3^ mm^2^/s, and 0.927 × 10^−3^ mm^2^/s, respectively. IVIM-DWI, intravoxel incoherent motion diffusion-weighted imaging; ADC, pure diffusion coefficient; f, perfusion fraction; D*, pseudo-diffusion coefficient; D, pure diffusion coefficient; NPC, nasopharyngeal carcinoma; T2WI, T2-weighted image.

**Figure 3:**
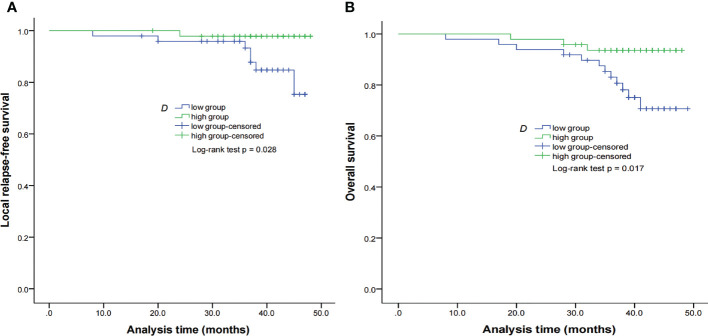
Kaplan–Meier curves of LRFS **(A)** and OS **(B)** for NPC patients stratified as the low D and high D groups. Low D group = patients with a primary lesion pretreatment D value ≤ 0.699 × 10^−3^ mm^2^/s; high D group = patients with a primary lesion pretreatment D value > 0.699 × 10^−3^ mm^2^/s. NPC, nasopharyngeal carcinoma; D, pure diffusion coefficient; LRFS, local relapse-free survival; OS, overall survival.

**Figure 4:**
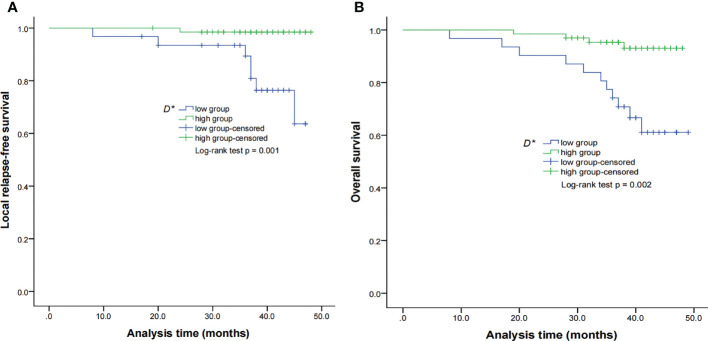
Kaplan–Meier curves of LRFS **(A)** and OS **(B)** for NPC patients stratified as the low D* and high D* groups. Low D* group = patients with a primary lesion pretreatment D* value ≤ 8.71 × 10^−3^ mm^2^/s; high D group = patients with a primary lesion pretreatment D value > 8.71 × 10^−3^ mm^2^/s. NPC, nasopharyngeal carcinoma; D*, pseudo-diffusion coefficient; LRFS, local relapse-free survival; OS, overall survival.

**Figure 5:**
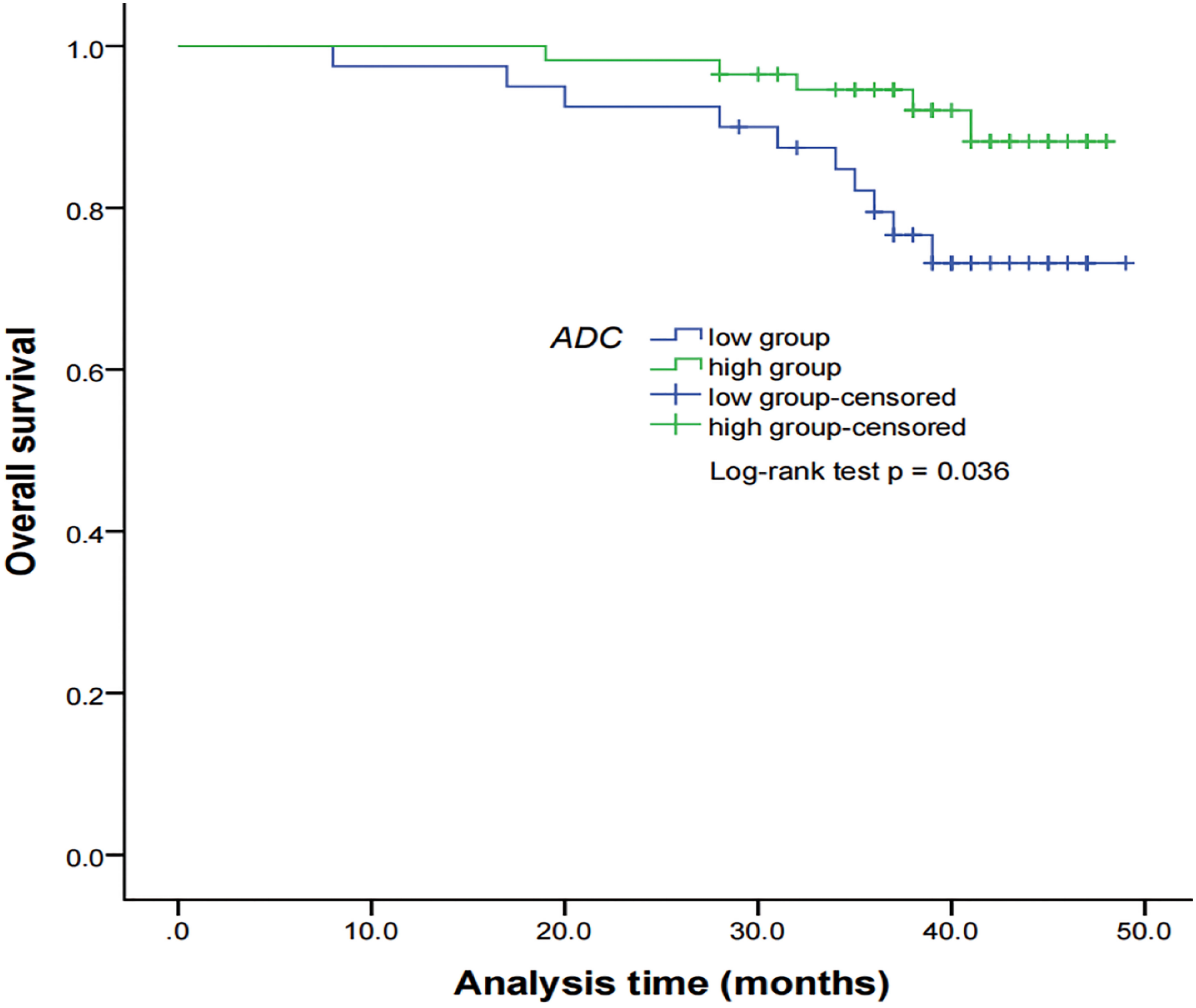
Kaplan–Meier curves of OS for NPC patients stratified as the low ADC and high ADC groups. Low ADC group = patients with a primary lesion pretreatment ADC value ≤ 0.897 × 10^−3^ mm^2^/s; high ADC group = patients with a primary lesion pretreatment ADC value > 0.897 × 10^−3^ mm^2^/s. NPC, nasopharyngeal carcinoma; ADC, apparent diffusion coefficient; OS, overall survival.

By using a non-parametric log-rank test, the optimal thresholds of the IVIM-DWI parameters for LRFS were set at 0.897 × 10^−3^ mm^2^/s for ADC (>0.897 × 10^−3^ mm^2^/s, n = 57; ≤0.897 × 10^−3^ mm^2^/s, n = 40), 0.699 × 10^−3^ mm^2^/s for D (>0.699 × 10^−3^ mm^2^/s, n = 48; ≤0.699 × 10^−3^ mm^2^/s, n = 49), 8.71 × 10^−3^ mm^2^/s for D* (>8.71 × 10^−3^ mm^2^/s, n = 66; ≤8.71 × 10^−3^ mm^2^/s, n = 31), and 0.198% for f (>0.198%, n = 73; ≤0.198%, n = 24). According to the results of the log-rank test, the higher ADC group demonstrated significantly higher OS rates than the low ADC group (*p* = 0.036), the higher D group showed significantly higher LRFS and OS rates than the low D group (*p* = 0.028 and *p* = 0.017, respectively), and the higher D* group exhibited significantly higher LRFS and OS than the lower D* group (*p* = 0.001 and *p* = 0.002, respectively). There were no significant differences in the LRFS, LRRFS, RRFS, DMFS, and PFS between the high ADC and low ADC groups; in the LRRFS, RRFS, DMFS, and PFS between the high D and low D groups; in the LRRFS, RRFS, DMFS, and PFS between the high D* and low D* groups; and in the LRRFS, LRFS, OS, RRFS, DMFS, and PFS between the high f and low f groups (all *p* > 0.05, **Table 3**). **Table 1** shows the numerical values of the parameters. IVIM parametric maps of ADC, D, D*, and f in two patients are illustrated in **Figure 2**. The Kaplan–Meier survival curves for LRFS and OS stratified by D, D*, and ADC are shown in **Figures 3A, B**, **4A, B**, and **5**, respectively.

**Table 3:** Univariate analyses of the prognostic factors in the 97 patients with nasopharyngeal carcinoma.

Endpoint	Variable	*p*
LRFS	ADC	0.062
	D D*	0.028* 0.001*
	f	0.257
LRRFS	ADC	0.335
	D D*	0.315 0.686
	f	0.388
RRFS	ADC	0.051
	D D*	0.063 0.125
	f	0.440
DMFS	ADC	0.769
	D D*	0.689 0.666
	f	0.723
PFS	ADC	0.380
	D D*	0.590 0.715
	f	0.972
OS	ADC	0.036*
	D D*	0.017* 0.002*
	f	0.300

LRFS, local relapse-free survival; LRRFS, local-regional relapse-free survival; RRFS, regional relapse-free survival; DMFS, distant metastasis-free survival; PFS, progression-free survival; OS, overall survival; ADC, apparent diffusion coefficient. *p<0.05.

### Independent prognostic parameters for nasopharyngeal carcinoma

When IVIM-DWI derived parameters, together with confounding factors (sex, age, clinical stage, treatment regimen, and pathological type), were calculated in multivariable Cox proportional hazards analysis, ADC and D were found to be the independent prognostic factors for LRFS (*p* = 0.041 and *p* = 0.037, respectively), D was found to be the independent prognostic factor for LRRFS (*p* = 0.045), D* and f were the independent prognostic factors for OS (*p* = 0.019 and 0.029, respectively), and f acted as an independent prognostic factor for DMFS (*p* = 0.020) (**Table 4**).

**Table 4:** Multivariate analyses of the prognostic factors in the 97 nasopharyngeal carcinoma patients.

Variable	RRFS			LRFS				LRRFS	
	*p*		HR (95% CI)	*p*		HR (95% CI)		*p*	HR (95% CI)
ADC	0.451		7.383 (0.037–9.916)	0.041		0.005 (0–2.373)		0.144	0.020 (0–3.849)
D D*	0.747 0.975		0.419 (0.002–2.910) 1.001 (0.942–1.064)	0.037 0.181		4.596 (4.070–8.973) 0.999 (0.942–1.060)		0.045 0.854	4.320 (0.551–7.685) 0.995 (0.946–1.047)
f	0.839		0.427 (0.001–6.296)	0.688		5.234 (0.002–16.836)		0.783	0.375 (0–0.762)
Age	0.944		1.003 (0.933–1.077)	0.304		1.042 (0.964–1.126)		0.897	0.996 (0.933–1.063)
Sex	0.802		1.227 (0.248–6.069)	0.353		1.025 (0.182–5.777)		0.991	1.008 (0.257–3.956)
T stage	0.827		1.688 (0.016–3.013)	0.397		2.683 (0.273–26.364)		0.565	2.248 (0.143–35.364)
N stage	0.748		2.157 (0.020–3.021)	0.853		0.807 (0.083–7.834)		0.515	0.376 (0.020–7.153)
M stage	0.250		0.210 (0.015–2.995)`	0.969		0 (0–0)		0.141	0.131 (0.009–1.956)
Overall stage	0.655		0.326 (0.002–4.657)	0.300		0.217 (0.012–3.906)		0.963	0.923 (0.031–27.126)
Pathological type	0.988		0 (0–0)	0.805		0.771 (0.098–6.049)		0.242	4.299 (0.373–49.532)
Treatment regimen	0.995		2.853 (0–4.786)	0.244		0.109 (0.003–4.523)		0.322	0.203 (0.009–4.762)
**Variable**	**RRFS**			**LRFS**				**LRRFS**	
	***p***		**HR (95% CI)**	***p***		**HR (95% CI)**		***p***	**HR (95% CI)**
ADC	0.339		0.014 (0–90.022)	0.072		0.060 (0.003–1.289)		0.871	0.560 (0.001–3.123)
D D*	0.685 0.019		7.274 (0.001–11.793) 0.647 (0.449–0.932)	0.060 0.903		1.225 (0.892–2.733) 0.998 (0.958–1.038)		0.746 0.913	3.682 (0.001–6.252) 0.997 (0.938–1.059)
f	0.029		0.004 (0–0.115)	0.387		0.076 (0–25.966)		0.020	0.002 (0–0.072)
Age	0.614		1.044 (0.884–1.233)	0.663		0.990 (0.944–1.037)		0.047	0.933 (0.872–0.999)
Sex	0.578		0.378 (0.012–11.724)	0.135		2.218 (0.780–6.307)		0.742	1.343 (0.231–7.793)
T stage	0.105		0.009 (0–2.638)	0.088		0.272 (0.061–1.215)		0.062	0.100 (0.009–1.123)
N stage	0.051		0.005 (0–0.294)	0.083		0.108 (0.009–1.341)		0.064	0.014 (0.001–0.266)
M stage	0.063		0.034 (0.001–1.202)	0.061		0.062 (0.011–0.337)		0.976	0 (0–0)
Overall stage	0.938		0.005 (0.202–9.479)	0.613		1.658 (0.233–11.783)		0.746	3.682 (0.001–13.123)
Pathological type	0.579		0.285 (0.003–23.888)	0.464		2.026 (0.307–13.371)		0.071	8.528 (1.972–74.099)
Treatment regimen	0.942		1.270 (0.002–8.954)	0.425		3.186 (0.185–54.800)		0.583	0.233 (0.001–42.096)

ADC, apparent diffusion coefficient; D, pure diffusion coefficient; D*, pseudo-diffusion coefficient; f, perfusion fraction; LRRFS, local-regional relapse-free survival; LRFS, local relapse-free survival; RRFS, regional relapse-free survival; HR, hazard ratio; CI, confidence interval.

## Discussion

Compared with traditional DWI models, the IVIM model fits signal attenuation in a bi-exponential decay mode, which enables its parameters to more accurately reflect water molecular diffusion and blood perfusion (19). In recent years, the IVIM model has been widely used to investigate renal perfusion and characterize prostate and breast cancers (23–25). In this study, we aimed to determine the feasibility of perfusion and diffusion parameters derived from IVIM-DWI in predicting the long-term outcome for NPC. Data from this study showed that baseline IVIM-DWI parameters D, D*, and ADC were associated with the prognosis of NPC, but f showed no relationship with the treatment outcome. We found that patients with lower ADC values demonstrated significantly lower OS rates than the patients with higher ADC values, the higher D group showed significantly higher LRFS and OS rates than the low D group, and the higher D* group exhibited significantly higher LRFS and OS than the lower D* group. Moreover, ADC and D acted as independent prognostic factors for LRFS, D was found to be the independent prognostic factor for LRRFS, D* and f were the independent prognostic factors for OS, and f acted as an independent prognostic factor for DMFS. However, the clinical characteristics failed to show a significant correlation with survival for NPC.

The potential of ADC in therapy assessment and prognosis prediction of NPC has been reported earlier (20, 21, 26–31). Several previous studies have shown that primary tumors with lower baseline ADC values had a better response to chemoradiotherapy as compared to those with higher baseline ADC values for NPC (32, 33). In regard to long-term outcomes, low pretreatment ADC was reported to be a good prognostic factor for shorter OS for patients with NPC in Yan’s study (29). Similar to the research, the results from our study showed that patients who had ADC value over a pre-specified cutoff had longer OS than those who had ADC value below the cutoff, which indicated that higher pretreatment ADC may be related to better long-term outcomes for NPC patients. The underlying mechanism for these similar findings may be that lower pretreatment ADC levels generally predict more aggressive biological features of tumors (33). These consistent findings suggest that tumors with high ADC values indicate low proliferation and consequently less cell subtype resistance to chemotherapy and thus respond better to chemoradiotherapy than tumors with low ADC values (33). However, contrary to our results, lower ADC value was found to be related to longer LRFS and DFS in NPC treated with chemoradiation (20, 31). Nevertheless, no significant relationship was demonstrated between pretreatment ADC and long-term outcomes in several studies (21, 30). These conflicting findings may be due to tumor heterogeneity and the differences in treatment regimens and imaging protocols across different studies.

The IVIM-DWI theory indicates that D is one of the diffusion-related parameters and corresponds to pure diffusion (34). Previous studies have reported that the low D values were related to high cell densities, indicating restricted Brownian motion of water (35–37). Similarly, the increase in cell density and different amounts of stromal tissues could reduce the molecular diffusivity (38), the same as the diffusion of oxygen, and lead to hypoxia, which can cause less sensitivity to radiotherapy and chemotherapy in NPC. In a previous study, lower D was proved to be significantly linked to poor treatment effect (39). Our study further certified that patients with low D values exhibited significantly lower LRFS and OS rates than the patients with high D values, which demonstrated that higher baseline D may be related to better survival for NPC patients. This may imply that tumors with low pretreatment diffusion values, indicating high cellularity and restricted diffusion, exhibited worse outcomes after chemoradiotherapy than tumors with high diffusion values, indicating low cellularity and much necrosis and cystic change. However, conflicting findings are present in the NPC literature for D, with studies showing the opposite results; that is, patients with low D values are related to better tumor response or long-term outcomes (21).

D* and f are both perfusion-related parameters derived from IVIM-DWI and depended on tumor microvascular attenuation. The D* value was defined according to the signal intensity ratios of the blood capillaries. Our data revealed that patients in the higher pretherapy D* value group showed significantly higher LRFS and OS rates than the low D* value group, which indicated that a higher D* value may imply better long-term outcomes in NPC. Similar to our observations, Chen et al. found that D* was significantly higher in the effective group than in the poor-effective group for NPC after treatment (39). These similar findings may be attributed to more neovascularization in tissues in the high D* group than in the low D* group. The new vessels can improve oxygenation by delivering blood and oxygen to the tumor lesions. Previous studies also have confirmed that high D* values have been shown to reflect neovascularization and increased tissue perfusion, and well-perfused tumors respond better to radiotherapy and chemotherapy (19). However, many other studies failed to show any relationship between D* and therapeutic effect evaluation and prognosis (17, 21). The differences in treatment regimens and time points selected for evaluating therapeutic response among these studies might account for the abovementioned inconsistent results.

As another perfusion-related parameter derived from IVIM-DWI, f showed no correlation with any of the survival endpoints in the present study. A similar consequence of f was previously reported in many other studies, which suggested that f was not a good predictor of treatment response and prognosis for NPC (11, 21, 40). The possible explanation for this result comes from prior studies that demonstrated the f value’s measurement was found to largely rely on the TE and the T2 relaxation time (41, 42). In addition, the technical restrictions of using f have been shown in prior investigations on head and neck cancer (14, 43, 44).

Our multivariable data showed that all the IVIM-DWI parameters were independent prognostic factors for survival in NPC, but the clinical factors including sex, age, clinical stage, treatment regimen, and pathological type were not, which implied that IVIM-DWI is superior to clinical factors in predicting the long-term outcome of NPC. In addition, the treatment regimen of NPC is mainly determined according to the clinical TNM stage at present, and only anatomical changes are considered. Therefore IVIM-DWI parameters have an advantage in predicting long-term outcomes in comparison with clinical factors because they are non-invasive.

This study has several limitations. First, the correlation between IVIM parameters and histologic characteristics was not measured in the study; this would help clarify the pathophysiologic sense of the IVIM parameters. Second, patients’ images were processed on a 1.5-T scanner in this study, which suggests that the spatial resolution was suboptimal, and it could be improved in further study by using 3.0- T scanners. Third, it is monocentric, and the cohort studied is relatively small, which may affect the analysis of TNM stages. Therefore, further studies with larger cohorts and analysis between IVIM-DWI parameters or texture features derived from IVIM-DWI and histologic characteristics are warranted to comprehensively understand the prognostic value of IVIM-DWI in NPC.

In conclusion, pretreatment IVIM-DWI parameters ADC, D, and D* could act as useful factors for predicting long-term outcomes and selecting high-risk patients with NPC.

## Data availability statement

The original contributions presented in the study are included in the article/supplementary material. Further inquiries can be directed to the corresponding author.

## Ethics statement

The studies involving human participants were reviewed and approved by the Institutional Ethics Review Committee of West China Hospital. The patients/participants provided their written informed consent to participate in this study.

## Author contributions

YQ and CC initiated this study, participated in its design, and performed study selection, data extraction, and data analysis. YQ drafted the manuscript. FG supervised all aspects of the study. HC revised the language. All authors contributed to the article and approved the submitted version.

## Funding

This study was supported by the National Natural Science Foundation of China (no. 81930046, 81771800, and 81829003), the State Key Project of Research and Development Plan of China (no. 2016YFA0201402), and the International Cooperation Project of Science and Technology Plan of Sichuan (no. 2017HH0045).

## Conflict of interest

The authors declare that the research was conducted in the absence of any commercial or financial relationships that could be construed as a potential conflict of interest.

## Publisher’s note

All claims expressed in this article are solely those of the authors and do not necessarily represent those of their affiliated organizations, or those of the publisher, the editors and the reviewers. Any product that may be evaluated in this article, or claim that may be made by its manufacturer, is not guaranteed or endorsed by the publisher.

## References

[1] Parkin DM Bray F Ferlay J Pisani P. Global cancer statistics. CA Cancer J Clin (2002) 55:74–108. doi: 10.3322/canjclin.55.2.74 15761078

[2] Lee AW Lin JC Ng WT. Current management of nasopharyngeal cancer. Semin Radiat Oncol (2012) 22(3):233–44. doi: 10.1016/j.semradonc.2012.03.008 22687948

[3] Pfister DG Spencer S Brizel DM Burtness B Busse PM Caudell JJ. Head and neck cancers, version 1.2015. J Natl Compr Canc Netw (2015) 13(7):847–55. doi: 10.6004/jnccn.2015.0102 PMC4976490 26150579

[4] Kam MK Wong FC Kwong DL Sze HC Lee AW. Current controversies in radiotherapy for nasopharyngeal carcinoma (NPC). Oral Oncol (2014) 50(10):907–12. doi: 10.1016/j.oraloncology.2013.09.013 24126221

[5] Sun X Su S Chen C Han F Zhao C Xiao W. Long-term outcomes of intensitymodulated radiotherapy for 868 patients with nasopharyngeal carcinoma: an analysis of survival and treatment toxicities. Radiother Oncol (2014) 110(3):398–403. doi: 10.1016/j.radonc.2013.10.020 24231245

[6] Xu T Zhu G He X Ying H Hu C. A phase III randomized study comparing neoadjuvant chemotherapy with concurrent chemotherapy combined with radiotherapy for locoregionally advanced nasopharyngeal carcinoma: updated long-term survival outcomes. Oral Oncol (2014) 50(2):71–6. doi: 10.1016/j.oraloncology.2013.11.002 24315404

[7] Shen T Tang L Luo D Chen Q Li P Mai D. Different prognostic values of plasma Epstein-Barr virus DNA and maximal standardized uptake value of 18F-FDG PET/CT for nasopharyngeal carcinoma patients with recurrence. PloS One (2015) 10(4):e0122756. doi: 10.1371/journal.pone.0122756 25853677 PMC4390333

[8] Kam MK Teo PM Chau RM Cheung KY Choi PH Kwan WH. Treatment of nasopharyngeal carcinoma with intensity-modulated radiotherapy: the Hong Kong experience. Int J Radiat Oncol Biol Phys (2004) 60:1440–50. doi: 10.1016/j.ijrobp.2004.05.022 15590175

[9] Lai V Li X Lee VH Lam KO Fong DY Huang B. Nasopharyngeal carcinoma: comparison of diffusion and perfusion characteristics between different tumour stages using intravoxel incoherent motion MR imaging. Eur Radiol (2014) 24(1):176–83. doi: 10.1007/s00330-013-2995-7 23990005

[10] Huang B Wong CS Whitcher B Kwong DL Lai V Chanet Q. Dynamic contrast-enhanced magnetic resonance imaging for characterising nasopharyngeal carcinoma: comparison of semiquantitative and quantitative parameters and correlation with tumour stage. Eur Radiol (2013) 23(6):1495–502. doi: 10.1007/s00330-012-2740-7 23377545

[11] Hou J Yu X Hu Y Li F Xiang W Wang L. Value of intravoxel incoherent motion and dynamic contrast-enhanced MRI for predicting the early and short-term responses to chemoradiotherapy in nasopharyngeal carcinoma. Med (Baltimore) (2016) 95(35):e4320. doi: 10.1097/MD.0000000000004320 PMC5008531 27583847

[12] Zheng D Chen Y Liu X Chen Y Xu L Wang R. Early response to chemoradiotherapy for nasopharyngeal carcinoma treatment: Value of dynamic contrast-enhanced 3.0 T MRI. J Magn Reson Imaging (2015) 41(6):1528–40. doi: 10.1002/jmri.24723 25136770

[13] Le Bihan D. Intravoxel incoherent motion perfusion MR imaging: a wake-up call. Radiology (2008) 249(3):748–52. doi: 10.1148/radiol.2493081301 19011179

[14] Yu X Hou J Li F Hu Y Lu Q Wang L. Intravoxel incoherent motion MRI for predicting early response to induction chemotherapy and chemoradiotherapy in patients with nasopharyngeal carcinoma. J Magn Reson Imaging (2016) 43(5):1179–90. doi: 10.1002/jmri.25075 26540374

[15] Xiao Y Pan J Chen Y Chen Y He Z Zheng X. Intravoxel incoherent motion-magnetic resonance imaging as an early predictor of treatment response to neoadjuvant chemotherapy in locoregionally advanced nasopharyngeal carcinoma. Med (Baltimore) (2015) 94(24):e973. doi: 10.1097/MD.0000000000000973 PMC4616555 26091468

[16] Paudyal R Oh JH Riaz N Venigalla P Li J Hatzoglou J. Intravoxel incoherent motion diffusion weighted MRI during chemoradiation therapy to characterize and monitor treatment response in human papillomavirus head and neck squamous cell carcinoma. J Magn Reson Imaging (2017) 45(4):1013–23. doi: 10.1002/jmri.25523 PMC5363344 27862553

[17] Xiao Y Chen Y Chen Y He Z Yao Y Pan J. Longitudinal assessment of intravoxel incoherent motion diffusion weighted imaging in evaluating the radio-sensitivity of nasopharyngeal carcinoma treated with intensity-modulated radiation therapy. Cancer Res Treat (2019) 51(1):345–56. doi: 10.4143/crt.2018.089 PMC6334000 29764118

[18] Yang X Wang Y Liang S He S Chen D Chen H. Comparison of the seventh and eighth editions of the UICC/AJCC staging system for nasopharyngeal carcinoma: analysis of 1317 patients treated with intensity-modulated radiotherapy at two centers. BMC Cancer (2018) 29;18(1):606. doi: 10.1186/s12885-018-4419-1 PMC5975550 29843648

[19] Bihan DL Breton E Lallemand D Aubin ML Vignaud J Laval-Jeantet M. Separation of diffusion and perfusion in intravoxel incoherent motion MR imaging. Radiology (1988) 168(2):497–505. doi: 10.1148/radiology.168.2.3393671 3393671

[20] Zhang Y Liu X Zhang Y Li W Chen L Mao Y. Prognostic value of the primary lesion apparent diffusion coefficient (ADC) in nasopharyngeal carcinoma: a retrospective study of 541 cases. Sci Rep (2015) 17; 5:12242. doi: 10.1038/srep12242 PMC4505330 26184509

[21] Qamar S King AD Ai Q So TY Mo FK Chen W. Pre-treatment intravoxel incoherent motion diffusion-weighted imaging predicts treatment outcome in nasopharyngeal carcinoma. Eur J Radiol (2020) 129:109127. doi: 10.1016/j.ejrad.2020.109127 32563165

[22] Li J Li W Niu J Song X Wu W Gong T. Intravoxel incoherent motion diffusion-weighted MRI of infiltrated marrow for predicting overall survival in newly diagnosed acute myeloid leukemia. Radiology (2020) 295(1):155–61. doi: 10.1148/radiol.2020191693 32068504

[23] Qi J Olsen NJ Price RR Winston JA Park JH. Diffusion-weighted imaging of inflammatory myopathies: polymyositis and dermatomyositis. J Magn Reson Imaging (2008) 27(1):212–7. doi: 10.1002/jmri.21209 18022843

[24] Riches SF Hawtin K Charles-Edwards EM Souza NM. Diffusion-weighted imaging of the prostate and rectal wall: comparison of biexponential and monoexponential modelled diffusion and associated perfusion coefficients. NMR BioMed (2009) 22(3):318–25. doi: 10.1002/nbm.1328 19009566

[25] Liu C Liang C Liu Z Zhang S Huang B. Intravoxel incoherent motion (IVIM) in evaluation of breast lesions: comparison with conventional DWI. Eur J Radiol (2013) 82(12):e782–9. doi: 10.1016/j.ejrad.2013.08.006 24034833

[26] Tangyoosuk T Lertbutsayanukul C Jittapiromsak N. Utility of diffusion-weighted magnetic resonance imaging in predicting the treatment response of nasopharyngeal carcinoma. Neuroradiol J (2021) 3:19714009211055191. doi: 10.1177/19714009211055191 PMC9437492 34730049

[27] Dong Z Andrews Z Xie C Yokoo T. Advances in MRI techniques and applications. BioMed Res Int (2015) 2015:139043. doi: 10.1155/2015/139043 26421276 PMC4572435

[28] Lu L Li Y Li W. The role of intravoxel incoherent motion MRI in predicting early treatment response to chemoradiation for metastatic lymph nodes in nasopharyngeal carcinoma. Adv Ther (2016) 33(7):1158–68. doi: 10.1007/s12325-016-0352-3 27294489

[29] Yan D Zhang W Ke S Zhao F Yan S Wang Q. The prognostic value of pretreatment tumor apparent diffusion coefficient values in nasopharyngeal carcinoma. BMC Cancer (2017) 11;17(1):678. doi: 10.1186/s12885-017-3658-x PMC5637091 29020937

[30] Law BK King AD Bhatia KS Ahuja AT Kam MK Ma BB. Diffusion-weighted imaging of nasopharyngeal carcinoma: Can pretreatment DWI predict local failure based on long-term outcome? AJNR Am J Neuroradiol (2016) 37(9):1706–12. doi: 10.3174/ajnr.A4792 PMC7984690 27151750

[31] Huang T Lu N Lian S Li H Yin S Geng Z. The primary lesion apparent diffusion coefficient is a prognostic factor for locoregionally advanced nasopharyngeal carcinoma: a retrospective study. BMC Cancer (2019) 17;19(1):470. doi: 10.1186/s12885-019-5684-3 PMC6525458 31101029

[32] Chen Y Liu Y Zheng D Xu L Hong L Xu Y. Diffusion-weighted magnetic resonance imaging for early response assessment of chemoradiotherapy in patients with nasopharyngeal carcinoma. Magn Reson Imaging (2014) 32(6):630–7. doi: 10.1016/j.mri.2014.02.009 24703576

[33] Zheng D Chen Y Chen Y Xu L Lin F Lin J. Early assessment of induction chemotherapy response of nasopharyngeal carcinoma by pretreatment diffusion-weighted magnetic resonance imaging. J Comput Assist Tomogr (2013) 37(5):673–80. doi: 10.1097/RCT.0b013e31829a2599 24045239

[34] Bihan DL Turner R. The capillary network: a link between IVIM and classical perfusion. Magn Reson Med (1992) 27(1):171–8. doi: 10.1002/mrm.1910270116 1435202

[35] Padhani AR. Diffusion magnetic resonance imaging in cancer patient management. Semin Radiat Oncol (2011) 21(2):119–40. doi: 10.1016/j.semradonc.2010.10.004 21356480

[36] Yankeelov TE Arlinghaus LR Li X Gore JC. The role of magnetic resonance imaging biomarkers in clinical trials of treatment response in cancer. Semin Oncol (2011) 38(1):16–25. doi: 10.1053/j.seminoncol.2010.11.007 21362513 PMC3073543

[37] Bonekamp S Corona-Villalobos CP Kamel IR. Oncologic applications of diffusion-weighted MRI in the body. J Magn Reson Imaging (2012) 35(2):257–79. doi: 10.1002/jmri.22786 22271274

[38] White ML Zhang Y Robinson RA. Evaluating tumors and tumorlike lesions of the nasal cavity, the paranasal sinuses, and the adjacent skull base with diffusion-weighted MRI. J Comput Assist Tomogr (2006) 30(3):490–5. doi: 10.1097/00004728-200605000-00023 16778627

[39] Chen WB Zhang B Liang L Dong YH Cai GH Liang CH. To predict the radiosensitivity of nasopharyngeal carcinoma using intravoxel incoherent motion MRI at 3.0 T. Oncotarget (2017) 21;8(32):53740–50. doi: 10.18632/oncotarget.17367 PMC5581146 28881847

[40] Qin Y Yu X Hou J Hu Y Li F Wen L. Predicting chemoradiotherapy response of nasopharyngeal carcinoma using texture features based on intravoxel incoherent motion diffusion-weighted imaging. Med (Baltimore) (2018) 97(30):e11676. doi: 10.1097/MD.0000000000011676 PMC6078652 30045324

[41] Sumi M Cauteren MV Sumi T Obara M Ichikawa Y Nakamura T. Salivary gland tumors: use of intravoxel incoherent motion MR imaging for assessment of diffusion and perfusion for the differentiation of benign from malignant tumors. Radiology (2012) 263(3):770–7. doi: 10.1148/radiol.12111248 22447854

[42] Lemke A Laun FB Simon D Stieltjes B Schad LR. An in vivo verification of the intravoxel incoherent motion effect in diffusion-weighted imaging of the abdomen. Magn Reson Med (2010) 64(6):1580–5. doi: 10.1002/mrm.22565 20665824

[43] Huang Y Chen T Zhang X Zeng N Li R Tang Y. Intravoxel incoherent motion diffusion-weighted imaging of resectable oesophageal squamous cell carcinoma: association with tumour stage. Br J Radiol (2018) 91(1084):20170421. doi: 10.1259/bjr.20170421 29308923 PMC5965982

[44] Kang KM Choi SH Kim DE Yun TJ Kim JH Sohn CH. Application of cardiac gating to improve the reproducibility of intravoxel incoherent motion measurements in the head and neck. Magn Reson Med Sci (2017) 10;16(3):190–202. doi: 10.2463/mrms.mp.2016-0051 PMC5600025 27818467

